# A unified benchmark of supervised and retrieval-based methods for viral genomic sequence classification

**DOI:** 10.1038/s41598-026-67272-9

**Published:** 2026-09-03

**Authors:** Ahmed M. Fahmy, Melissa Ayad, Hassan M. Ahmed

**Affiliations:** 1 Center for Informatics Science (CIS), School of Information Technology and Computer Science (ITCS), Nile University, Giza, Egypt; 2 Département d’informatique, Faculté des Sciences, Université de Sherbrooke, Québec, Canada; 3 Department of Biomedical Engineering, Faculty of Engineering, Capital University (formerly Helwan University), Cairo, Egypt

**Keywords:** Genomic sequence classification, Genotyping, Dense retrieval, Vector search, Approximate nearest neighbor search, Genomic image processing, Computational biology and bioinformatics, Mathematics and computing

## Abstract

The rapid growth of genomic sequencing demands fast, accurate, and scalable analysis methods. In viral genomic classification, expanding labeled reference collections can make supervised models costly to update and dependent on fixed label sets, motivating retrieval-based genomic classification as a simpler, more flexible alternative. We present a unified benchmark of supervised and retrieval-based methods for viral genomic sequence classification across three viral classification tasks: hepatitis C virus (HCV) genotyping, COVID-19 discrimination, and human papillomavirus (HPV) genotyping. We compare standard sequence encodings (one-hot, *k*-mers, FCGR) with dense embeddings (dna2vec, DNABERT). For each representation, we evaluate supervised classifiers (Random Forest, Decision Tree, XGBoost) and retrieval-based classification, where sequence vectors are indexed with FAISS and labels are assigned via similarity-weighted k-NN. Furthermore, we benchmark multiple FAISS index types (Flat, IVF, HNSW, IVFPQ, OPQ) to characterize accuracy-speed–memory trade-offs at scale. The results show that XGBoost and retrieval using Flat or IVF indexes achieve strong classification performance under different computational profiles. Compressed indexes such as IVFPQ and OPQ substantially reduce memory usage, although their accuracy loss depends on the dataset and representation. Overall, supervised XGBoost provides a favorable accuracy–size trade-off, while retrieval-based classification remains competitive and allows labeled reference sequences to be incorporated without retraining a global classifier. This benchmark provides practical guidance for selecting sequence representations, classifiers, and vector-search indexes under different accuracy, memory, and update requirements.

**Supplementary Information:**

The online version contains supplementary material available at https://doi.org/10.1038/s41598-026-67272-9.

##  Introduction and background

   Genotype classification is a fundamental task in genomics, underpinning the identification of evolutionary relationships, functional elements, and genetic variants in biological sequences^1^. However, traditional approaches for DNA sequence classification often rely on labor-intensive sequence annotation or pairwise alignment, which become impractical as genomic datasets grow in size and diversity^2^. The growing volume of sequencing data produced by modern technologies demands efficient computational methods to analyze and categorize genotypes at scale. Alignment-based tools like BLAST^3^ are effective for finding close matches, yet they struggle with distant homology and have high computational costs on large databases. Such limitations have motivated the development of alignment-free classification techniques that can leverage sequence characteristics without explicit sequence alignment^4^. Alignment-free sequence analyses have been applied to problems ranging from whole-genome phylogeny^5^ to protein family classification^6^, highlighting their broad utility in analyzing genomic data.

A key challenge in computational genomic analysis is how to represent DNA sequences in a machine-readable form that preserves biologically relevant information. To this end, various sequence encoding strategies have been proposed to capture the features of DNA as an input into machine learning models^7,8^. A straightforward classical method is one-hot encoding, which represents each nucleotide (A, C, G, T) as a categorical indicator. An alternative classical approach uses *k*-mer frequency vectors. *k*-mer counting or bag-of-words models treat each sequence as a document of *k*-mers (words) and characterize it by the frequencies of the possible k-length substrings.

Beyond linear *k*-mer models, more efficient encoding techniques leverage the spatial or graphical representation of sequences. Amongst them is the Chaos Game Representation (CGR) which maps a one-dimensional DNA sequence into a two-dimensional fractal image^9^. In a CGR plot, each point is iteratively placed to encode *k*-mer composition, resulting in a unique image (genomic fingerprint) for each sequence. A quantitative variant, the Frequency Chaos Game Representation (FCGR), divides the CGR image into a grid and computes the frequency of *k*-mers in each cell, yielding a fixed-size numeric matrix (or image) for a given sequence^10^. These alignment-free encodings provide diverse ways to numerically describe genomic sequences, and comparing them can reveal which characteristics of the sequences are most informative for distinguishing genetic classes.

In recent years, deep learning embeddings have emerged as powerful sequence representations, inspired by advances in natural language processing^11,12^. Rather than handcrafted or basic features, deep models learn to encode sequences into dense vector representations that capture underlying patterns and context. One approach is dna2vec, which extends the idea of Word2Vec to DNA *k*-mers: it trains a neural network on large genomic corpora to embed *k*-mers such that those appearing in similar contexts have similar vector representations^13^. The resulting embeddings have been shown to correlate with biological similarity measures.

Recent genomic language models adopt different tokenization and sequence-modeling strategies. DNABERT uses fixed-length *k*-mer tokenization^14^, whereas DNABERT2 employs byte-pair encoding (BPE)^15^. Other models, such as HyenaDNA^16^ and Caduceus^17^, operate at single-nucleotide resolution, while Nucleotide Transformer^18^ and MetaBERT^19^ provide additional pretrained genomic representation-learning approaches. Related studies have also explored learned sequence representations and embedding-based prediction in other biological settings. Soylu and Sefer^20^ employed a BERT-based architecture for biological sequence prediction, while Sefer and Kingsford^21^ investigated metric and semimetric embeddings for biological annotation prediction. These studies further demonstrate the broader utility of learned representations and embedding-based approaches for biological sequence analysis.

Dense retrieval treats genotype assignment as a search problem rather than a purely supervised prediction task. Dense retrieval involves embedding sequences into a vector space and using similarity search to find the nearest neighbors or most similar sequences for a given query. If an encoding is biologically meaningful, sequences from the same genotype (or class) should cluster together in the embedding space, allowing retrieval-based classification—essentially, classification by finding the closest known sequences. In applications like lineage-assignment, models generally require regular updates as new sequences and labels accumulate: their accuracy degrades when query genomes drift away from the training distribution, forcing frequent retraining on expanding reference sets. Toole et al.^22^ report that the pangoLEARN classifier’s performance drops for genomes that differ substantially from its training data, necessitating “regular and frequent” retraining on updated lists of designated sequences^22^.

Recent genomic studies have started to adopt vector search frameworks for this purpose, leveraging tools like Facebook’s FAISS library for fast similarity search^23^. For example, Refahi et al.^2^ embedded gene sequences with a transformer model and indexed them with FAISS to enable efficient similarity queries across large sequence collections, demonstrating efficient detection of novel sequences and taxonomic classification via embedding comparisons. This paradigm affords scalability where new labeled sequences can be incorporated by re-indexing rather than retraining. It may also support more transparent decision-making through the inspection of retrieved reference examples.

In this study, we conduct a comprehensive comparison of different representation schemes—one-hot, bag-of-*k*-mers, FCGR, and dense embeddings—across two task families: (1) supervised classification using Random Forest, Decision Tree, and XGBoost; and (2) similarity-based classification (classification-by-retrieval) with FAISS. Although XGBoost, Random Forest, and Decision Tree are commonly associated with tabular data, the sequence representations evaluated in this study transform variable-length genomic sequences into fixed-length numerical feature vectors.

For example, k-mer and FCGR representations encode sequence composition as structured numerical features, while dna2vec and DNABERT produce fixed-dimensional dense embeddings. Consequently, these representations can be directly used as inputs to conventional supervised classifiers. Tree-based models were included as computationally efficient and widely used baselines for different sequence representations under a classification setting.

For similarity-based classification, we evaluate multiple index families in FAISS: Flat (exact brute-force), Inverted File index (IVF), Hierarchical Navigable Small World graph (HNSW), Inverted File with Product Quantization (IVFPQ), and Optimized Product Quantization (OPQ).

In addition to classification accuracy, we conduct detailed analyses of computational efficiency, including model (index) size, training and inference time, and search latency. We also study how the length of the sequence representation impacts resource requirements. This comprehensive setup enables a systematic comparison of learning paradigms and encoding methods under a unified lens of accuracy, memory, and runtime efficiency.

The reported runtime and memory differences should be interpreted relative to the scale of the datasets evaluated in this benchmark. At this scale, small absolute differences in execution time or memory consumption may have limited practical significance. Their operational importance is expected to become more relevant for substantially larger reference collections, and dedicated large-scale scalability experiments are therefore required before drawing strong conclusions about deployment-level efficiency.

The present benchmark does not aim to exhaustively compare all available DNA language models. DNABERT was selected as a representative transformer-based genomic embedding model to provide a contrast with classical sequence representations and dna2vec. More recent models, including DNABERT2, Nucleotide Transformer, HyenaDNA, and Caduceus, employ different tokenization or sequence-modeling strategies and constitute important directions for future benchmarking.

Similarly, the reported model and index sizes should be interpreted as relative resource measurements within the present experimental scale. Small absolute differences in model size may have limited practical importance for the datasets considered here, and their operational relevance would need to be evaluated on substantially larger reference collections.

It is important to mention that similarity-based sequence search, nearest-neighbor classification, and embedding-based retrieval are established computational concepts. Accordingly, the contribution of this study is a controlled and unified empirical benchmark that evaluates how multiple sequence representations, supervised classifiers, and FAISS-based retrieval indexes behave under common viral genomic classification settings.

We summarize our contribution as follows:

1. *Unified benchmark across representations and tasks.* We conduct a controlled, head-to-head comparison of four encoding families—one-hot, bag-of-*k*-mers, FCGR, and dense embeddings (dna2vec, DNABERT)—across three genotyping problems (HCV, human coronavirus, and HPV classification tasks), evaluated under both supervised and retrieval-based classification.
2. *Dual evaluation protocol.* We assess supervised classifiers (Random Forest, Decision Tree, XGBoost) alongside similarity-based classification (k-NN over FAISS), outlining regimes in which retrieval matches or exceeds trained models.
3. *Vector-search index space evaluation.* We systematically examine different FAISS index families: Flat, IVF, HNSW, IVFPQ, and OPQ—quantifying accuracy–speed trade-offs via index-build time and query latency.
4. *Practical and encoder-agnostic evaluation pipeline.* We provide a reproducible workflow for generating sequence representations, training supervised classifiers, building vector indexes, and evaluating predictive and computational trade-offs.

## Research methodology

   The research methodology centers around evaluating genotype classification using similarity-based retrieval techniques. Rather than relying solely on traditional supervised classification, the study frames the task as a dense retrieval problem—treating genotype prediction as finding the most similar sequences in a high-dimensional embedding space. We use a pipeline where genomic sequences are first encoded using various methods (e.g., one-hot, *k*-mer, FCGR, and deep embeddings). These encoded vectors are then used to build a searchable vector store. For any new query sequence, the same encoding is applied, and similarity search (via FAISS) is performed to retrieve the nearest neighbors. The final genotype prediction is made through k-nearest neighbor (k-NN) voting, leveraging similarity scores to weight class votes. This retrieval-based classification approach is benchmarked against supervised machine learning models using multiple genomic datasets (HCV, COVID-19, and HPV) to evaluate predictive accuracy and computational efficiency.

**Table 1:** Sequence counts per genotype grouped by dataset.

Dataset	Genotype	Count
HCV	hcv_1a	10001
	hcv_1b	10001
	hcv_3a	10001
	hcv_4	6516
	hcv_6	5415
	hcv_2a	2781
	hcv_2b	2327
	hcv_3b	1582
	hcv_5	1466
	hcv_2c	1002
HPV	hpv_16	8227
	hpv_31	2690
	hpv_18	1055
	hpv_58	1048
	hpv_11	294
COVID-19	SARS-CoV-2	3700
	HCoV-OC43	1351
	MERS-CoV	734
	HCoV-NL63	637
	HCoV-229E	465
	HCoV-HKU1	412
	SARS-CoV-1	59

### Dataset

   We conducted the evaluations on three curated genomics corpora: (i) HCV genotypes, (ii) Human coronaviruses (HCoV) framed as a binary task, and (iii) HPV genotypes. We include three viral datasets (HCV, COVID-19, HPV) instead of a single benchmark to cover different tasks (binary vs multi-class), virus families (RNA vs DNA), and dataset scales. The class distributions for the three datasets are presented in Table 1.

For the HCV dataset, genomic sequences were obtained from the Los Alamos Hepatitis C Sequence Database^24,25^. The collection contains both complete and partial genomes spanning ten genotypes (1a, 1b, 2a, 2b, 2c, 3a, 3b, 4, 5, and 6). Following the binary formulation used in previous work^26^, the human coronavirus dataset was divided into two classes: COVID-19, comprising SARS-CoV-2 sequences, and non-COVID-19, comprising the remaining human coronavirus sequences (HCoV-HKU1, HCoV-NL63, MERS-CoV, HCoV-OC43, SARS-CoV-1, and HCoV-229E). For brevity, this binary classification problem is referred to as the COVID-19 task throughout the manuscript. Whole and partial genomes for the seven human coronaviruses were downloaded from NCBI: HCoV-229E, HCoV-HKU1, HCoV-NL63, HCoV-OC43, MERS-CoV, SARS-CoV-1, and SARS-CoV-2. For HPV, sequences were collected from NCBI for five target genotypes: HPV-11, HPV-16, HPV-18, HPV-31, and HPV-58.

For preprocessing, we applied a uniform preprocessing procedure to all datasets. Sequences were converted to uppercase and restricted to the canonical nucleotide alphabet $\{A,C,G,T\}$; all other symbols were removed.

Exact duplicate sequences were removed before data partitioning, retaining only the first occurrence. Deduplication was therefore performed across the complete dataset rather than independently within the training and test partitions. The datasets contained both complete and partial genomic sequences. These records were retained because the objective was to evaluate classification under heterogeneous sequence-length conditions.

**Fig. 1:**
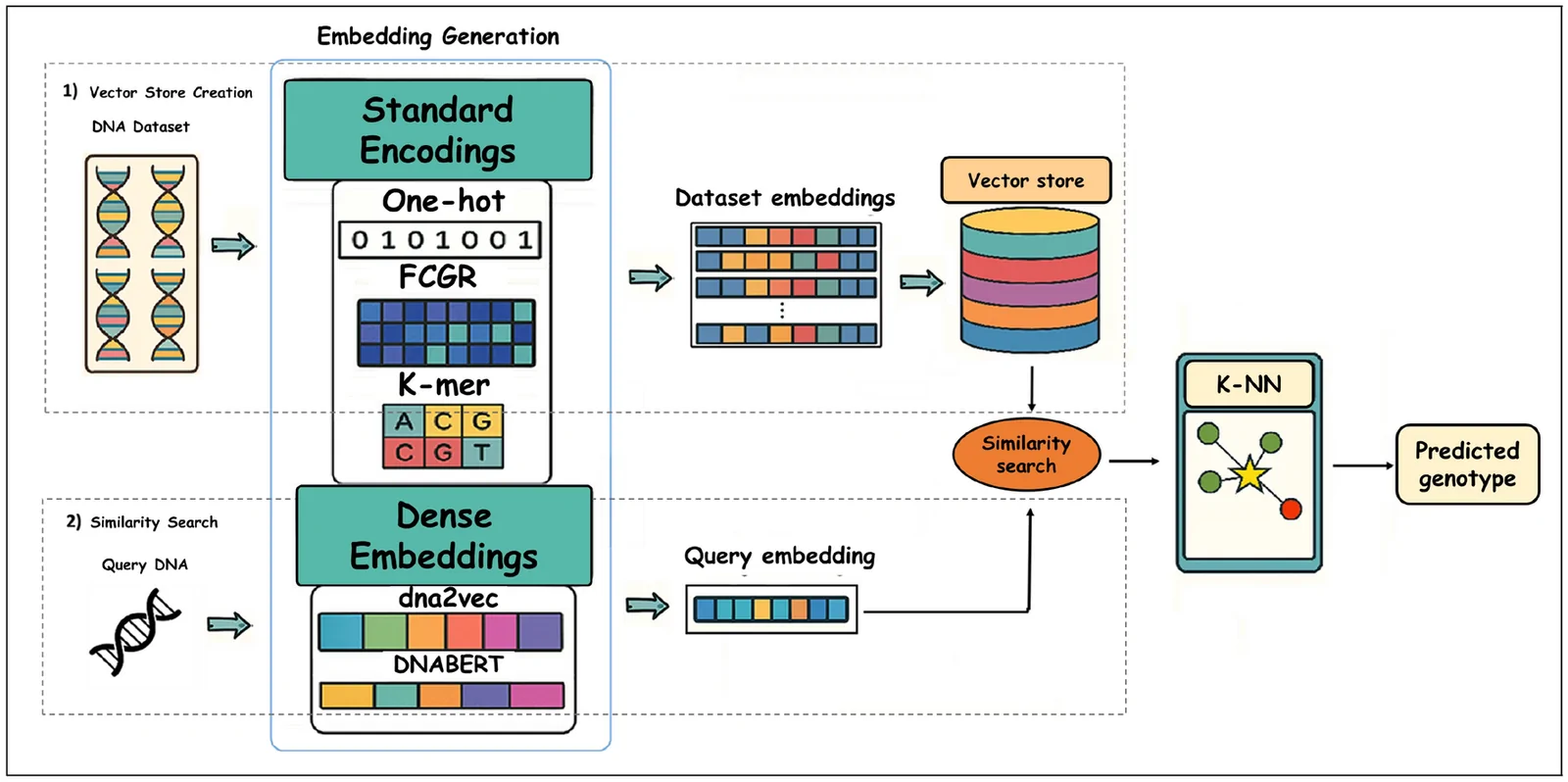
Overview of the similarity-based classification pipeline. Training sequences are encoded (standard: one-hot, FCGR, *k*-mer; or dense: dna2vec, DNABERT) to build a vector store. A query is encoded the same way, then nearest neighbors are retrieved from the vector store and k-NN voting yields the predicted genotype.

### Similarity-based classification

   We cast the genotype prediction task as nearest-neighbor retrieval in an embedding space (Fig. 1). Let *f(· )* denote an encoder that maps a DNA sequence *s* to a vector $x=f(s)\in \mathbb {R}^d$ (used encoders are detailed in Section Encoding methods). During preparation, every training sequence $s_i$ is embedded to $x_i$ and paired with its label $y_i$. All vectors are $\ell _2$-normalized,

1 $$\begin{aligned} \tilde{x} \;=\; \frac{x}{\Vert x\Vert _2}, \end{aligned}$$

At inference, a query sequence $s_q$ is encoded to $\tilde{x}_q$. We then retrieve its *k* nearest neighbors under cosine similarity $\operatorname {sim}(\tilde{x}_q, \tilde{x}_i), \text {where } \operatorname {sim}(\tilde{x}_q, \tilde{x}_i) \in [-1, 1].$

Efficient search is performed with FAISS; index choices (Flat, IVF, HNSW, IVFPQ, OPQ) and their trade-offs are detailed in Section Indexing for similarity search. For a query *q*, the search returns the neighbor index list and its aligned similarity scores:

2 $$\begin{aligned} \mathcal {N}_k(q) = [\, i_1, i_2, \dots , i_k \,], \qquad \textbf{w}(q) = [\, w_{i_1}, w_{i_2}, \dots , w_{i_k} \,]. \end{aligned}$$

Here, $w_{i_j}=\operatorname {sim}(\tilde{x}_q,\tilde{x}_{i_j})$, $\mathcal {N}_k(q)$ denotes the indices of the *k* closest points to *q* in the database, and $\textbf{w}(q)$ contains the similarity scores between *q* and each neighbor. Equivalently, we can write the paired output as $\{(i_1,w_{i_1}),\dots ,(i_k,w_{i_k})\}$. To convert neighbors into a class prediction, we use similarity-weighted voting. For each label *c* among the *k* neighbors, we accumulate

3 $$\begin{aligned} V(c)\;=\;\sum _{i\in \mathcal {N}_k(q)\,:\,y_i=c} w_i, \qquad \hat{y}_q \;=\; \arg \max _{c} V(c). \end{aligned}$$

Here, *V*(*c*) denotes the sum of similarities of all neighbors whose label is *c*, and the predicted class $\hat{y}_q$ is chosen as the one with the highest aggregated vote. This rule is parameter-free, and leverages the graded evidence carried by the retrieved similarities. For example, let *k=3*. For a query *q*, vector store returns the neighbor indices and aligned cosine similarities

$$. \mathcal {N}_3(q) = [\, 42,\, 7,\, 105 \,], \qquad \textbf{w}(q) = [\, 0.81,\, 0.76,\, 0.10 \,]. $$

Assume the corresponding labels are

$$ y_{42}=\texttt {G3},\quad y_{7}=\texttt {G1},\quad y_{105}=\texttt {G3}. $$

Similarity–weighted votes per class are

$$ V(\texttt {G3}) = 0.81 + 0.10 = 0.91,\qquad V(\texttt {G1}) = 0.76. $$

Hence the prediction is

$$ \hat{y}_q \;=\; \arg \max _{c} V(c) \;=\; \texttt {G3}. $$

### Indexing for similarity search

   Modern similarity search over large collections of embeddings requires data structures that make nearest-neighbor queries fast without exhaustively scanning every vector. Indexing refers to building such structures to help in partitioning the space, organizing points, or compressing them, so that at query time we examine only a small candidate set while still retrieving neighbors that are close under a chosen metric (e.g., cosine similarity). Because of the curse of dimensionality, modern similarity search relies on Approximate Nearest Neighbor (ANN) indexes to achieve sub-linear query times by trading off a small amount of accuracy for significant speed gains^27^.

Instead of a one-size-fits-all approach, FAISS offers multiple index types (each with distinct data structures and algorithms) to balance speed, memory usage, and accuracy under different conditions. In this study, we evaluate five index types used in our methodology (Flat, IVF, HNSW, IVFPQ, and OPQ) explaining their conceptual workings, indexing mechanisms, theoretical trade-offs, and suitable usage scenarios. All five methods operate in the context of high-dimensional vector embeddings, with the goal of quickly finding points that are nearest to a query vector under a given distance metric.

**Fig. 2:**
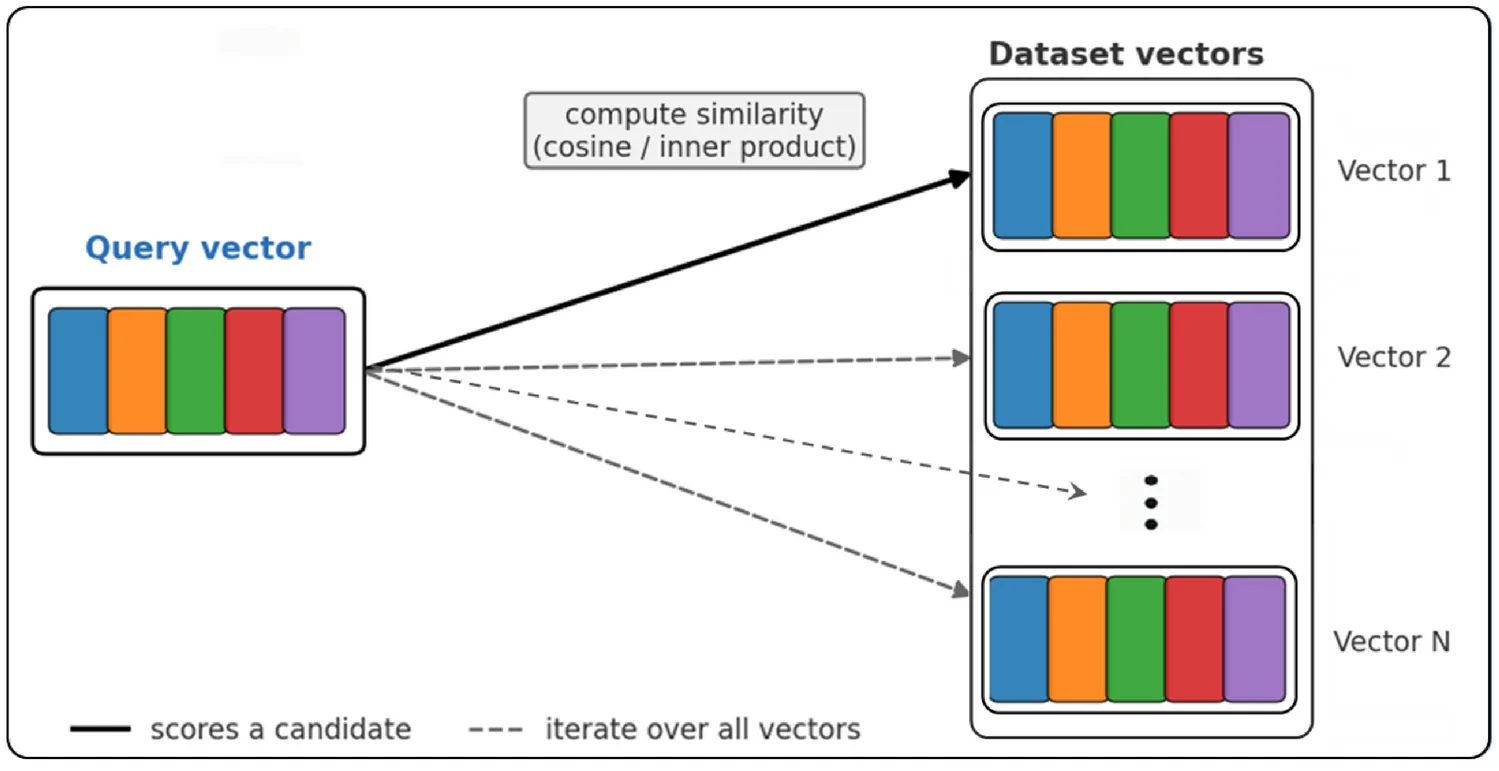
The brute-force approach to vector similarity search using a Flat index. A query vector is compared against all dataset vectors by computing similarity.

#### Flat index (exhaustive search)

   The Flat index (also known as IndexFlat in FAISS) is the simplest baseline, which performs a brute-force linear scan of all vectors to find exact nearest neighbors. Figure 2 demonstrates the brute-force approach.

Conceptually, the index stores the full dataset of vectors in an array and computes the distance from the query to every vector in turn, returning the closest matches. This exhaustive approach guarantees the highest accuracy (it finds the true nearest neighbors) since no approximation is introduced. The implementation is straightforward and no special data structure or training is required beyond storing the raw vectors. However, the Flat index incurs linear time complexity $\mathcal {O}(N)$ per query (where *N* is the number of stored vectors), which becomes a bottleneck as datasets grow large. In high-dimensional spaces, computing millions of distances for each query is computationally expensive and does not scale to real-time systems.

In summary, Flat indexing is well-suited for small datasets (tens of thousands) or strict accuracy requirements, for example when N is small or as a ground-truth baseline. In cases where absolute recall is needed and query latency is less critical, the Flat index provides a simple solution free of approximation error.

#### Inverted file index

**Fig. 3:**
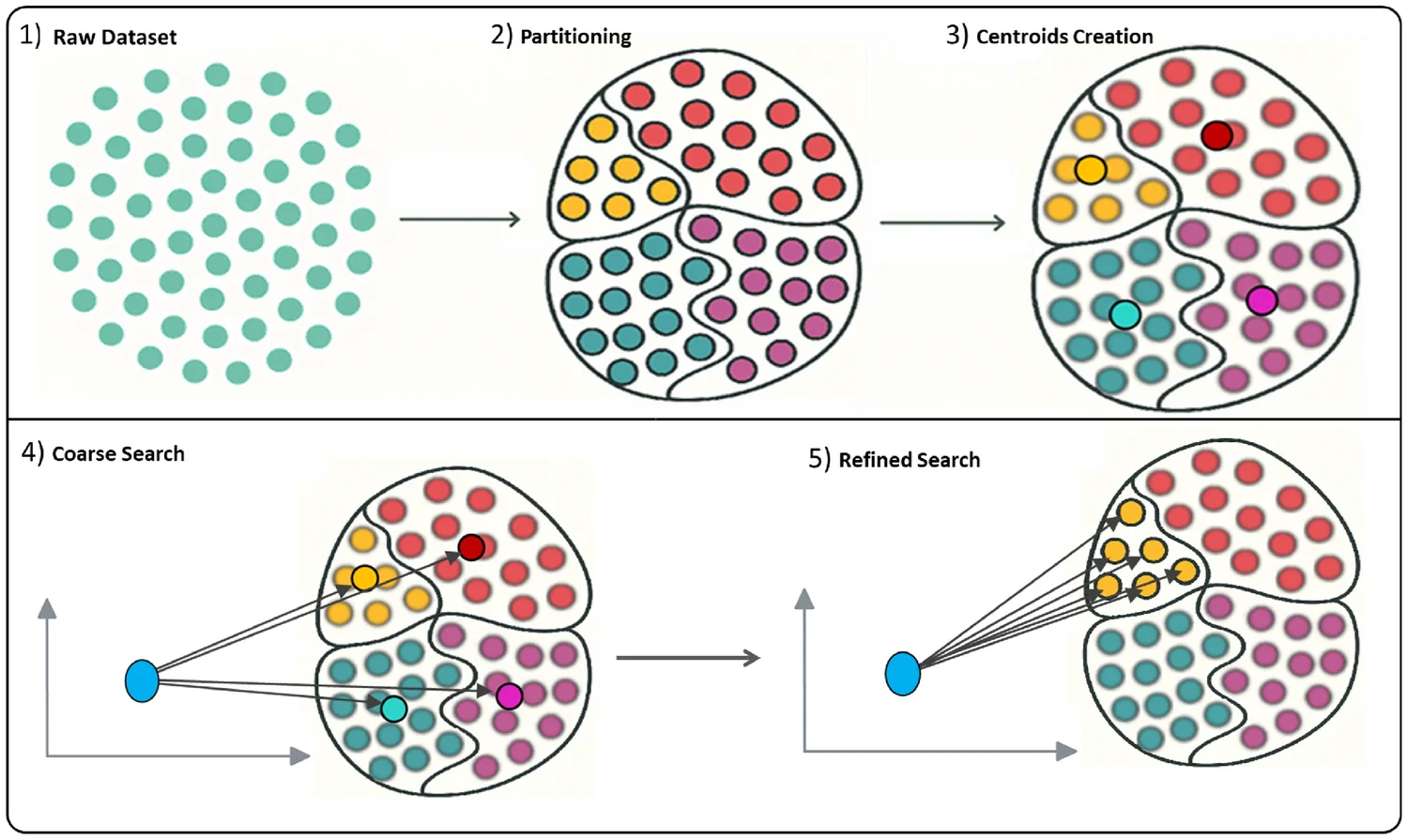
Illustration of the Inverted File (IVF) index search process: (1) Start with a raw dataset, (2) partition it into clusters, (3) compute centroids for each cluster, (4) perform a coarse search by comparing the query to centroids, and (5) conduct a refined search within the nearest cluster to retrieve relevant results efficiently.

   The IVF index is a classic vector indexing approach that accelerates search by partitioning the data space into clusters and limiting searches to a few relevant clusters. Figure 3 shows the IVF indexing process. During index construction, a coarse quantizer (e.g. k-means) is applied to the dataset to produce K cluster centroids, which partition the space into Voronoi cells. Each data vector is assigned to its nearest centroid, and vectors are stored in lists (inverted lists) associated with these centroids. At query time, the query vector is first compared to the K centroids, and the search is then directed to the vectors in the nearest few clusters instead of the entire dataset. By avoiding comparisons with vectors in distant clusters, IVF achieves a substantial speed-up over brute force. The advantage of IVF comes from reducing the search space per query. If vectors are evenly divided among K clusters, searching P clusters means the algorithm compares the query to roughly $\approx \frac{p}{K}\,N$ vectors instead of N.

The trade-off is that if a query’s true nearest neighbor lies outside the searched clusters, the result will be approximate. Memory-wise, IVF adds overhead for storing the centroid positions and the cluster listings, but this overhead is modest (centroids scale with K). In summary, IVF may be suitable for large-scale datasets for which exhaustive search is computationally expensive, offering a trade-off between search efficiency and retrieval accuracy.

#### Hierarchical navigable small world

   The HNSW index is a graph-based ANN structure that organizes vectors in a navigable small-world graph with multiple hierarchical layers. HNSW organizes the dataset as a layered, hierarchical graph. A small subset of nodes is probabilistically promoted to higher, sparser layers, where they act as entry hubs with long-range connections across the space; lower layers contain denser, short-range links. This structure enables fast hierarchical search with progressive refinement where queries start at a high layer to traverse large distances quickly and then descend to refine the search near the target region.

**Fig. 4:**
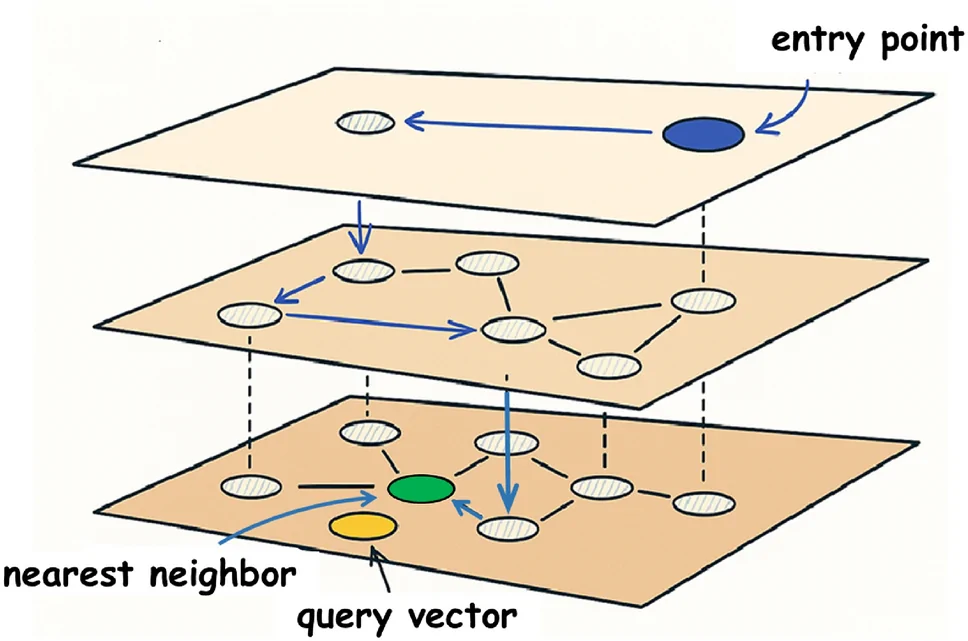
Illustration of the HNSW search process. The search begins from an entry point (blue) in the upper layer and progressively traverses connected nodes across lower graph layers toward the query vector (yellow), ultimately identifying its nearest neighbor (green).

Figure 4 visualizes the HNSW search process. Searching an HNSW graph is a greedy graph traversal augmented with a priority queue. The query starts at the top layer’s entry node and computes distances to that node’s neighbors, iteratively moving to the neighbor that is closest to the query. Once it reaches a local minimum on a given layer, the algorithm switches to the next lower layer, initializing the search there with the best candidate found so far. This process repeats until the bottom layer is reached, where the graph is densest; at the bottom layer a final greedy search finds the nearest neighbors of the query.

The advantages of HNSW lie in its excellent recall-speed balance and scalability on high-dimensional data. It is often the top-performing ANN method for many benchmarks, capable of reaching near exact recall with sufficient search effort, while still being much faster than brute force. The primary trade-off is memory footprint: storing graph neighbors (edges) for each node increases memory usage significantly. For instance, if each node stores 32 or 64 neighbors, the index can require several times the memory of the raw vectors. Additionally, construction of the graph can be time-consuming for very large *N*.

HNSW is ideal for large-scale, high-dimensional datasets where high recall is needed with low latency and one can afford a larger memory budget for the index. In scenarios where memory is constrained or data is relatively low-dimensional, other structures (like IVF or compressed indexes) might be preferred.

#### Inverted file index with product quantization

   The IVFPQ index combines the IVF clustering approach with Product Quantization (PQ) compression to achieve both search acceleration and memory reduction. In an IVFPQ index, the dataset is first partitioned into K clusters as in a standard IVF index. Then, instead of storing full vectors in each inverted list, each vector is compressed by PQ: the vector is divided into m subvectors, and each subvector is encoded as the index of the nearest centroid in a smaller codebook trained for that subspace. Thus, each original vector is represented by an m-tuple of centroid indices (a compact code). Equivalently, PQ produces a set of m codebooks (each with K entries, typically 256 entries for 8-bit codes) such that any vector can be approximated by a concatenation of one code from each codebook. Storing an m-component code (often just a few bytes, e.g. 8 or 16 bytes total) per vector yields a dramatic memory savings compared to storing 32-bit float coordinates for each dimension.

IVFPQ shines in extremely large-scale settings (e.g. millions to billions of vectors) where memory is at a premium and some loss in precision is acceptable for huge gains in efficiency. By compressing vectors, IVFPQ can fit massive databases in memory, and query times can be cut by orders of magnitude compared to uncompressed IVF.

#### Optimized product quantization

   The OPQ technique improves upon standard PQ by learning an optimal linear transformation of the vector space prior to quantization. The intuition is that by rotating or remapping the original feature space, one can better align it with the subspace partitioning used in PQ, thereby reducing quantization error. In vanilla PQ, the vector’s m subvectors are chosen by splitting the dimensions (often contiguous blocks of coordinates); this might be suboptimal if the data’s variance is not evenly distributed or if features are correlated. OPQ addresses this by finding a rotation (orthonormal matrix R) that decorrelates the components of each subvector and equalizes their variances. After applying this learned transform to all vectors, PQ codebooks are trained on the transformed data. This often yields a lower reconstruction error: essentially, OPQ tries to ensure that each PQ subquantizer is responsible for an equally “difficult” portion of the data, rather than, say, one subvector capturing most of the signal energy. The result is that for the same code length, OPQ achieves higher accuracy (lower distortion) than an un-optimized PQ.

OPQ is ideal when you need high-recall ANN at tight memory budgets because it improves PQ accuracy without growing codes. At query time it’s essentially as fast as IVFPQ; at build time there’s an offline training cost to learn the rotation. Selecting an index is a trade-off that depends on dataset scale, memory constraints, latency targets, and tolerance for approximation.

### Encoding methods

   A fundamental step in genomic data analysis is the transformation of raw nucleotide sequences into meaningful numerical representations that can be processed by machine learning and similarity-based algorithms. This study explores a range of encoding techniques, ranging from traditional representations to modern data-driven embeddings, to assess their effectiveness in downstream genotype classification tasks.

Standard methods include one-hot encoding, which maps each nucleotide to a binary vector; *k*-mer encoding, which captures local sequence patterns through fixed-length substrings; and FCGR, which encodes *k*-mer frequencies into spatial matrices. In contrast, dense embeddings like dna2vec and DNABERT use neural models to learn continuous vector representations from large DNA corpora, capturing contextual and biological patterns. These diverse encodings are evaluated for their impact on classification and retrieval performance.

#### One-hot encoding

   One-hot encoding is a simple and widely used method for representing nucleotide sequences as numerical vectors while preserving the exact identity of each base. In this encoding scheme, each symbol from the nucleotide alphabet $\Sigma = \{ A, C, G, T \}$, is assigned a fixed position in the vocabulary. The size of the vocabulary is *|V| = 4* as per $\{A, C, G, T\}$. For each nucleotide in the input sequence, we place its corresponding one-hot representation at its respective position in the sequence. More concretely, for a sequence $s = s_1 s_2 \dots s_L$ of length *L*, we build a binary matrix of shape *|V| × L*. Each column of the matrix represents a specific position in the input sequence, while each row of a given input position corresponds to one nucleotide in the predefined vocabulary. For example, given the input sequence $s = \texttt {ATG}$, the one-hot encoding is represented as a *4 × 3* matrix, where each column corresponds to a position in the sequence:

$$ M = \begin{bmatrix} 1 & 0 & 0 \\ 0 & 0 & 0 \\ 0 & 0 & 1 \\ 0 & 1 & 0 \end{bmatrix} $$

In this case, column 1 corresponds to A, so the entry for *A* is 1, the same for other columns. The same logic is applied to the remaining columns, where each column activates only the row corresponding to the nucleotide at that position.

#### *k*-mer encoding

   *k*-mer encoding represents the input sequence $s = s_{1}s_{2}\dots s_{L},\quad s_{i} \in \Sigma$ (typically $\Sigma = \{A, C, G, T\}$), as a multiset of all contiguous substrings of length *k* (*k*-mer).

Given a sequence $s=s_{1}\ldots s_{L}$, we form the overlapping kmer output as

4 $$\begin{aligned} \mathcal {K}_{k}(s) = \big (s_{1:k},\, s_{2:k+1},\, \ldots ,\, s_{L-k+1:L}\big ), \end{aligned}$$

with stride of 1 unless stated otherwise. For vocabulary construction, we fix a vocabulary $V=\Sigma ^{k}=\{t_{1},\ldots ,t_{|V|}\}$ with a stable column index per *k*-mer. The dimensionality is $|V|=4^{k}$. For example, assume *k=3* and the input sequence $s=\texttt {ACGTAC} \quad (L=6).$ The overlapping 3-mer stream is

$$ \mathcal {K}_3(s)=\big (\texttt {ACG},\texttt {CGT},\texttt {GTA},\texttt {TAC}\big ). $$

The full vocabulary is $V=\Sigma ^3$ with $|V|=4^3=64$. The entries in this representation follow lexicographic order: $V = (\texttt {AAA}, \texttt {AAC}, \ldots , \texttt {ACG}, \ldots , \texttt {TTT})$. Such a structured representation is particularly useful when applying machine learning models, as it guarantees alignment between features and biological motifs, enabling efficient computation and meaningful comparison. This encoding effectively preserves genomic patterns that are essential for distinguishing genotypic variations^28^. Fixed-length *k*-mer tokenization is simple and biologically intuitive, but it can be sensitive to local sequence variations because a single nucleotide change may affect several overlapping *k*-mers. Alternative tokenization strategies, such as byte-pair encoding (BPE), can provide more flexible subsequence representations and are used by newer genomic language models such as DNABERT2.

#### Frequency chaos game representation

   The FCGR is a numerical encoding method that transforms biological sequences, such as DNA or RNA, into a fixed-length representation that captures both the frequency distribution and spatial organization of *k*-mers. Originally derived from Chaos Game Representation (CGR), FCGR combines fractal geometry with frequency analysis, making it a powerful tool for computational genomics, including sequence classification, virus subtyping, and phylogenetic analysis.

In FCGR, each nucleotide in the alphabet $\{A, C, G, T\}$ is mapped to a unique corner of a unit square according to a binary coding scheme:

$$ A \rightarrow 00,\quad C \rightarrow 01,\quad T \rightarrow 10, \quad G \rightarrow 11. $$

Each *k*-mer is interpreted as a base-4 integer code. The unit square is recursively divided into $4^k$ subregions, resulting in a $2^k \times 2^k$ grid. Each unique *k*-mer corresponds to one specific cell in this grid.

For a given DNA sequence *S* of length *L*, a sliding window of size *k* is moved along the sequence to extract all possible *k*-mers. For each extracted *k*-mer, its corresponding cell in the grid is incremented by one. This yields a frequency matrix *F*, where

5 $$\begin{aligned} F(i,j) = \text {count of the { k}-mer mapped to cell } (i,j). \end{aligned}$$

**Fig. 5:**
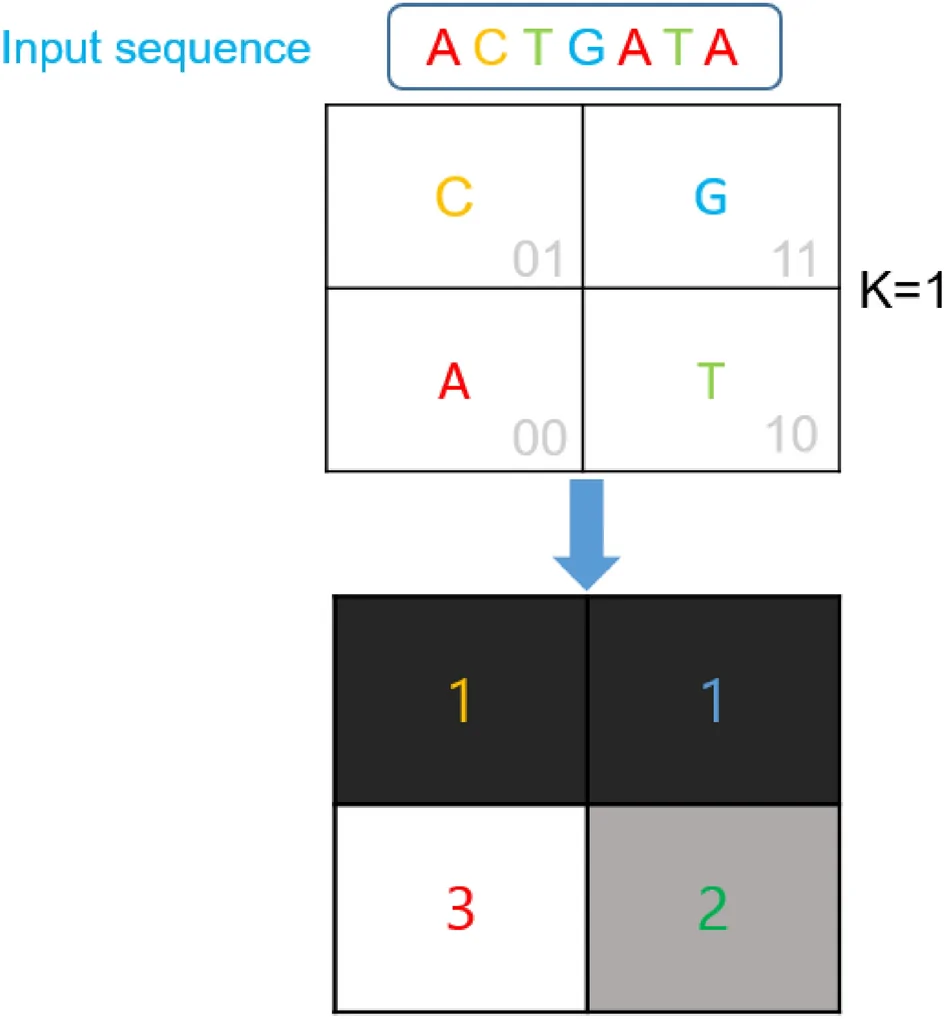
Illustration of nucleotide frequency mapping using *FCGR* encoding with *k=1*.

Figure 5 shows a simple example of FCGR encoding for *k=1* applied to the input DNA sequence *ACTGATA*. We can see that each nucleotide in $\mathcal {A} = \{A, C, G, T\}$ is mapped to a unique cell in a *2 × 2* grid according to its binary code. We then count the occurrence of each nucleotide in the sequence and place the resulting counts in their corresponding grid cells, resulting in a frequency matrix. This grid can then be either flattened into a one-dimensional feature vector or used directly as a 2D representation for further processing.

In our case, to help in the computations of the downstream machine learning and similarity tasks, the FCGR matrix is flattened into a one-dimensional vector of length $4^k$.

#### Dense embeddings

   Dense embeddings provide continuous representations of genomic sequences, enabling downstream models to exploit similarity and contextual patterns beyond sparse encodings. We consider two complementary families: (i) dna2vec, and (ii) DNABERT. Both approaches yield fixed-dimensional vectors per sequence and integrate seamlessly into similarity search and classification pipelines. To respect encoder context limits while preserving coverage on long inputs, we apply a sliding-window scheme with *25%* overlap: for a window length *W* and stride $S=\lfloor 0.75\,W\rfloor$, a sequence *s* is partitioned into overlapping windows $(w_1,\dots ,w_m)$ that together span the full sequence, including the tail.

Each window is encoded with either the dna2vec encoder or DNABERT to produce a single vector per window. The sequence representation is then formed by taking the uniform mean of its window vectors, yielding one fixed-dimensional embedding per sequence. We subsequently apply row-wise $\ell _2$ normalization so that inner-product ranking coincides with cosine similarity.

## Results and discussion

In this section, we evaluate how different sequence encodings, learning paradigms, and indexing strategies impact the accuracy, efficiency, and scalability of genomic sequence classification.

### Experimental setup

   This study introduces a unified experimental benchmark designed to assess both the effectiveness and efficiency of genomic classification models. All experiments were conducted using Google Colab^29^ with 32 GB of system RAM. The available NVIDIA L2 GPU was used exclusively to generate the DNABERT and dna2vec embeddings. All sequence preprocessing, traditional encoding, supervised model training, FAISS index construction, similarity search, and performance evaluation were performed on the CPU. We consider three genomic classification problems: HCV, COVID-19, and HPV genotyping.

For the evaluation strategy, each dataset was divided into stratified training and test partitions using a ratio of 80/20. Stratification was performed using the genotype or class label so that the class proportions were approximately preserved in both partitions. The individual sequence record was the splitting unit. All experiments were evaluated using exactly the same partitions to ensure comparability. Because each configuration was evaluated using a single fixed partition, small differences between closely performing methods should be interpreted descriptively rather than as evidence of statistically significant superiority.

For retrieval-based classification, all feature vectors were converted to 32-bit floating-point format and $\ell _2$-normalized, allowing inner-product search to be interpreted as cosine-similarity ranking. The evaluated FAISS index structures and their corresponding hyperparameters are summarized in Table 12 in Appendix A.

Representation-generation time was measured separately from downstream classification and retrieval operations. For supervised models, training time encompassed only model fitting, whereas prediction time corresponded to inference over the complete test set. For retrieval methods, index-construction time included index training, when required, and insertion of the training vectors. Search time included only the FAISS search operation; voting and metric computation were excluded. Accordingly, the reported training, prediction, indexing, and search times do not include data loading or representation generation.

Supervised model sizes were determined by serializing the fitted estimators using the highest available Python pickle protocol. FAISS index sizes were measured from their serialized representations. Peak Python-managed memory during model fitting was estimated using tracemalloc. These measurements do not represent total system memory usage because allocations made by compiled libraries and GPU operations may not be captured. The implementation was developed in Python 3.12 using NumPy, pandas, SciPy, scikit-learn, XGBoost, PyTorch, Hugging Face Transformers, and FAISS.

We investigate two broad families of representations. The first consists of standard encodings, and the second family comprises dense embeddings obtained from pre-trained models, namely dna2vec and DNABERT. Each encoding is evaluated on two tasks: supervised and similarity-based classification. For supervised classification, we train conventional models—Random Forest, Decision Tree, and XGBoost—on the resulting feature vectors. For similarity-based classification, we apply a classification-by-retrieval paradigm, where L2-normalized feature vectors are indexed in a FAISS-based vector store and labels are predicted by weighted voting over the top-*k* nearest neighbors. We study several FAISS index types (Flat, IVF, HNSW, IVFPQ, OPQ) to probe the trade-offs between accuracy, indexing cost, and query latency.

Across our configurations, we report both predictive accuracy and computational efficiency. For supervised models, we measure training time, prediction time, per-sample latency, and model size. For similarity-based classification, we record index-build time, and total search time. This unified setup allows a direct comparison of encoding, learning paradigms, and indexing schemes under consistent experimental conditions.

### Supervised classification performance

   In these experiments, we carry out a comparative evaluation of three classifiers—XGBoost, Random Forest, and Decision Tree—across the three genomic datasets. The classification performance is assessed using different sequence encoding strategies: one-hot encoding, *k*-mer encoding, and FCGR for standard representations as well as dna2vec and DNABERT for dense representations.

#### Classification over traditional encoders

   As shown in Tables 2, 3 and 4, across the traditional representations, XGBoost and Random Forest consistently outperformed Decision Tree, with the performance gap being most pronounced for HCV. Increasing the *k*-mer or FCGR order generally improved performance on HPV and HCV, although the gains became progressively smaller at higher orders. In contrast, the COVID-19 task remained close to ceiling performance across several configurations. Overall, *k*-mer and FCGR representations provided stronger supervised performance than one-hot encoding, indicating that composition-based representations were better suited to the classification tasks considered here.

**Table 2:** Classification accuracy of one-hot encoding across three genomic datasets.

Model	HPV	COVID-19	HCV
XGBoost	**0.969**	0.969	**0.962**
Random Forest	0.966	**0.973**	0.959
Decision Tree	0.944	0.949	0.910

Best result per dataset is **bolded**.

**Table 3:** Classification accuracy by model using *k*-mer encoding across the three genomic datasets.

Model	*k*-mer	HPV	COVID-19	HCV
XGBoost	3	0.9880	0.9973	0.9804
	4	0.9884	0.9973	0.9882
	5	0.9899	0.9964	0.9908
	6	**0.9914**	0.9946	**0.9910**
Random Forest	3	0.9884	**0.9991**	0.9758
	4	0.9902	0.9973	0.9841
	5	0.9910	0.9937	0.9880
	6	0.9906	0.9955	0.9897
Decision Tree	3	0.9658	0.9910	0.8461
	4	0.9722	0.9928	0.8702
	5	0.9801	0.9892	0.8963
	6	0.9809	0.9901	0.9202

Best result per dataset is **bolded**.

**Table 4:** Classification accuracy by model using FCGR representation across the three genomic datasets.

Model	FCGR-k	HPV	COVID-19	HCV
XGBoost	3	0.9820	**0.9973**	0.9837
	4	0.9835	0.9964	0.9897
	5	0.9854	0.9964	**0.9922**
	6	0.9865	0.9955	0.9919
Random Forest	3	0.9839	**0.9973**	0.9773
	4	0.9846	0.9955	0.9855
	5	0.9854	0.9964	0.9893
	6	**0.9869**	0.9964	0.9917
Decision Tree	3	0.9591	0.9883	0.8567
	4	0.9711	0.9910	0.8713
	5	0.9715	0.9919	0.8983
	6	0.9756	0.9901	0.9203

Best result per dataset is **bolded**.

#### Classification over dense embeddings

**Table 5:** Classification accuracy by model using dna2vec embeddings across the three genomic datasets.

Model	HPV	COVID-19	HCV
XGBoost	**0.9884**	**0.9928**	**0.9761**
Random Forest	0.9827	0.9919	0.9401
Decision Tree	0.9572	0.9884	0.7102

Best result per dataset is **bolded**.

**Table 6:** Classification accuracy by model using DNABERT embeddings across the three genomic datasets.

Model	HPV	COVID-19	HCV
XGBoost	**0.9849**	**1.000**	**0.9787**
Random Forest	0.9820	0.9973	0.9689
Decision Tree	0.9580	0.9937	0.8360

Best result per dataset is **bolded**.

   Tables 5 and 6 present the classification accuracy of the supervised models trained on dna2vec and DNABERT embeddings, respectively. With dense embeddings, XGBoost remained the strongest supervised classifier across the three datasets. DNABERT achieved higher performance than dna2vec on the COVID-19 and HCV tasks, whereas dna2vec performed slightly better on HPV. Importantly, the dense representations did not consistently outperform the simpler *k*-mer and FCGR representations. This indicates that the additional representational complexity of pretrained genomic embeddings does not necessarily translate into higher classification accuracy. The corresponding Macro-F1 scores for the supervised classification approach are reported in the Appendix B.1 for completeness.

### Similarity-based classification performance

In these experiments, we analyze the impact of input representation (One-hot, *k*-mer, FCGR, dna2vec, and DNABERT) and FAISS index structures (Flat, IVF, HNSW, IVFPQ, OPQ) on similarity-based classification accuracy across the three genomic datasets. Tables 7, 8, and 9 report the best accuracy achieved for each configuration, along with the optimal number of neighbors *k*.

**Table 7:** Similarity-based accuracy for One-hot representations with FAISS, over index types. For each dataset, we report the accuracy and the *k*-NN value that yields this best accuracy.

Index	HPV	COVID-19	HCV
Flat	0.9595 (k=3)	**0.9740** (k=1)	0.9598 (k=1)
IVF	**0.9606** (k=3)	**0.9740** (k=1)	**0.9601** (k=1)
HNSW	0.9591 (k=1)	0.9731 (k=1)	0.9591 (k=1)
IVFPQ	0.9384 (k=3)	0.9489 (k=1)	0.7183 (k=1)
OPQ	0.9598 (k=1)	0.9651 (k=1)	0.9368 (k=1)

Best result per dataset is **bolded**.

#### Vector similarity over traditional encoders

   As shown in Table 7, the performance of one-hot encodings is generally lower than learned or compositional representations. The Flat, IVF and HNSW indexes perform comparably and achieve the highest accuracy for most datasets.

For HPV and COVID-19, IVF and Flat yield the top scores (e.g., 0.9606 and 0.9740), indicating that exhaustive or coarse quantization still preserves sufficient neighbor structure. For HCV, performance slightly drops, and compressed indexes like IVFPQ severely underperform (e.g., 0.7183), showing that sparsity of one-hot vectors is not well preserved under quantization. Generally, OPQ and HNSW maintain moderate accuracy but show no clear advantage over simpler structures.

**Table 8:** Similarity-based accuracy for *k*-mer representations with FAISS, over index types and *k*-mer orders. For each dataset, we report the accuracy and the k-NN value that yields the best accuracy.

Index	*k*-mer	HPV	COVID-19	HCV
Flat	3	0.9887 (k=1)	0.9973 (k=1)	0.9863 (k=1)
	4	0.9910 (k=1)	**0.9982** (k=1)	0.9910 (k=1)
	5	0.9940 (k=3)	**0.9982** (k=1)	0.9917 (k=1)
	6	0.9940 (k=3)	**0.9982** (k=1)	0.9919 (k=5)
IVF	3	0.9880 (k=3)	0.9973 (k=1)	0.9861 (k=1)
	4	0.9910 (k=3)	**0.9982** (k=1)	0.9908 (k=1)
	5	**0.9944** (k=3)	**0.9982** (k=1)	0.9916 (k=1)
	6	0.9932 (k=5)	**0.9982** (k=1)	**0.9919** (k=1)
HNSW	3	0.9861 (k=1)	0.9973 (k=1)	0.9847 (k=1)
	4	0.9869 (k=1)	**0.9982** (k=1)	0.9871 (k=1)
	5	0.9861 (k=1)	**0.9982** (k=1)	0.9897 (k=1)
	6	0.9891 (k=1)	**0.9982** (k=1)	0.9912 (k=1)
IVFPQ	3	0.9857 (k=3)	0.9973 (k=1)	0.9741 (k=5)
	4	0.9902 (k=1)	**0.9982** (k=1)	0.9790 (k=5)
	5	0.9940 (k=1)	**0.9982** (k=1)	0.9740 (k=1)
	6	0.9936 (k=1)	**0.9982** (k=1)	0.9673 (k=5)
OPQ	3	0.9726 (k=3)	0.9973 (k=1)	0.9141 (k=5)
	4	0.9869 (k=5)	**0.9982** (k=1)	0.9195 (k=3)
	5	0.9910 (k=3)	**0.9982** (k=1)	0.9232 (k=5)
	6	0.9925 (k=3)	**0.9982** (k=1)	0.9291 (k=3)

Best result per dataset is **bolded**.

Table 8 reveals that *k*-mer representations significantly improve accuracy across all datasets and index types. For COVID-19, accuracy reaches nearly perfect scores (0.9982) consistently across all representations and indexes. For HPV and HCV, the Flat and IVF indexes achieve the highest accuracies (e.g., 0.9944 for HPV, 0.9919 for HCV) at *k=5* or *k=6*. For indexes IVFPQ and OPQ, despite compression, they still yield strong performance, particularly on COVID-19. However, accuracy deteriorated more markedly for HCV, particularly with IVFPQ and OPQ. Overall, k-mer representations offer a robust balance between retrieval performance and flexibility across index types, with lower *k*-NN values consistently sufficient for accurate classification.

**Table 9:** Similarity-based accuracy for each FAISS index type and FCGR order *k*. For each dataset, we report the accuracy and the *k*-NN value that yields this best accuracy.

Index	FCGR-*k*	HPV	COVID-19	HCV
Flat	3	0.9895 (k=1)	0.9973 (k=1)	0.9863 (k=1)
	4	0.9917 (k=1)	**0.9982** (k=1)	0.9908 (k=1)
	5	**0.9940** (k=3)	**0.9982** (k=1)	0.9914 (k=1)
	6	0.9936 (k=3)	**0.9982** (k=1)	**0.9923** (k=1)
IVF	3	0.9887 (k=1)	0.9973 (k=1)	0.9863 (k=1)
	4	0.9914 (k=1)	**0.9982** (k=1)	0.9903 (k=1)
	5	0.9936 (k=3)	**0.9982** (k=1)	0.9912 (k=1)
	6	0.9936 (k=3)	**0.9982** (k=1)	**0.9923** (k=1)
HNSW	3	0.9865 (k=1)	0.9973 (k=1)	0.9849 (k=1)
	4	0.9887 (k=1)	**0.9982** (k=1)	0.9866 (k=1)
	5	0.9884 (k=1)	**0.9982** (k=1)	0.9904 (k=1)
	6	0.9895 (k=1)	**0.9982** (k=1)	0.9912 (k=1)
IVFPQ	3	0.9861 (k=5)	0.9973 (k=3)	0.9743 (k=5)
	4	0.9895 (k=3)	**0.9982** (k=1)	0.9716 (k=5)
	5	0.9929 (k=3)	**0.9982** (k=1)	0.9686 (k=5)
	6	**0.9940** (k=3)	**0.9982** (k=1)	0.9651 (k=3)
OPQ	3	0.9854 (k=3)	**0.9982** (k=1)	0.9685 (k=3)
	4	0.9906 (k=1)	**0.9982** (k=1)	0.9780 (k=5)
	5	0.9932 (k=5)	**0.9982** (k=1)	0.9789 (k=3)
	6	0.9929 (k=3)	**0.9982** (k=1)	0.9816 (k=5)

Best result per dataset is **bolded**.

FCGR encodings, shown in Table 9, match or slightly exceed the performance of *k*-mer representations in many cases. For HPV and COVID-19, accuracy peaks at 0.9940–0.9982 for Flat and IVF indexes, comparable to *k*-mer results. For HCV, FCGR achieves the highest score among all encodings (up to 0.9923), particularly with Flat and IVF indexes at *k=6*. The compressed indexes, IVFPQ and OPQ, performed better with FCGR than with one-hot, especially on HCV, where OPQ reaches 0.9816 at *k=6*, confirming that FCGR is more compression-tolerant.

Overall, the effect of approximation and compression was strongly dependent on both the dataset and the representation. *k*-mer and FCGR were considerably more robust to compression than one-hot encoding, whereas the larger degradation observed for HCV indicates that aggressive vector compression can remove information relevant to finer-grained genotype discrimination. Most high-performing configurations also required only a small number of neighbors, suggesting that useful class information was concentrated within local neighborhoods of the representation space.

**Table 10:** Similarity-based accuracy for each FAISS index type and dna2vec embedding. For each dataset, we report the accuracy and the *k*-NN value that yields this best accuracy.

Index	HPV	COVID-19	HCV
Flat	**0.9914** (k=1)	**0.9973** (k=1)	**0.9876** (k=1)
IVF	**0.9914** (k=1)	**0.9973** (k=1)	0.9874 (k=1)
HNSW	0.9895 (k=1)	0.9946 (k=3)	**0.9876** (k=1)
IVFPQ	0.9846 (k=3)	0.9910 (k=1)	0.9270 (k=3)
OPQ	0.9854 (k=5)	0.9910 (k=1)	0.9690 (k=5)

Best result per dataset is **bolded**.

**Table 11:** Similarity-based accuracy for each FAISS index type and DNABERT embedding. For each dataset, we report the accuracy and the *k*-NN value that yields this best accuracy.

Index	HPV	COVID-19	HCV
Flat	**0.9846** (k=3)	**0.9982** (k=1)	**0.9790** (k=1)
IVF	0.9842 (k=3)	0.9973 (k=1)	0.9787 (k=1)
HNSW	0.9812 (k=3)	0.9973 (k=1)	0.9776 (k=1)
IVFPQ	0.9741 (k=5)	0.9973 (k=1)	0.8846 (k=5)
OPQ	0.9756 (k=5)	0.9964 (k=1)	0.9065 (k=5)

Significant values are in bold.

#### Similarity-based classification using dense embeddings

Tables 10 and 11 summarize retrieval performance using dna2vec and DNABERT embeddings, respectively. For both representations, Flat, IVF, and HNSW achieve closely comparable accuracy, indicating that moderate approximation generally preserves the relevant neighborhood structure.

In contrast, the quantization-based IVFPQ and OPQ indexes show larger performance losses, particularly for HCV, indicating that compression can remove information important for finer-grained genotype discrimination. Across the two embedding families, neither representation consistently dominates all datasets, although DNABERT performs slightly better on COVID-19 while dna2vec is marginally stronger on HPV and HCV in several configurations. High-performing results are generally obtained with small neighborhood sizes, with the optimal value depending on the dataset, representation, and index type. This indicates that class information is concentrated within local regions of the embedding space. The corresponding Macro-F1 scores for the similarity-based classification approach are reported in the Appendix B.2 for completeness.

### Comparative study of performance-resource trade-off

   In this section, we conduct a comprehensive analysis of model performance in relation to key resource constraints, namely time, memory, and representation complexity. Specifically, we compare supervised and retrieval-based approaches across the three genomic datasets, focusing on how each method balances classification accuracy with (i) training and inference time, (ii) memory footprint, and the length of input representations with respect to time complexity. This analysis aims to uncover practical trade-offs and guide the selection of models or indexing strategies based on task requirements, computational budgets, and scalability considerations.

#### Time-accuracy trade-off

**Fig. 6:**
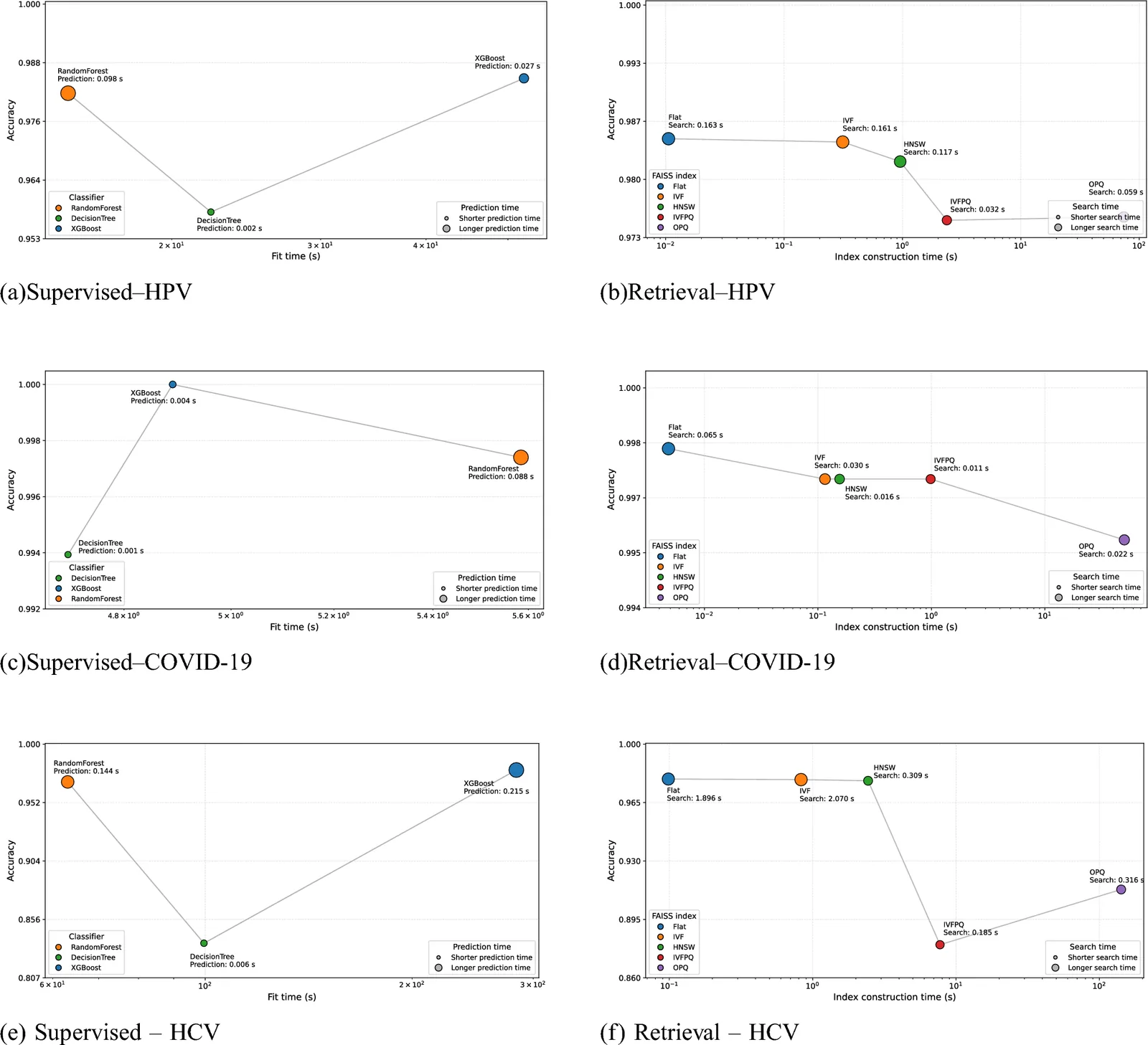
Comparison of classification accuracy and computational time for supervised and retrieval-based classification using DNABERT embeddings across the HPV, COVID-19, and HCV datasets. Each row compares the supervised and retrieval-based approaches for the same dataset.

   To assess the trade-offs between supervised and retrieval-based classification approaches on genomic data, we analyzed both paradigms using DNABERT embeddings across the HPV, COVID-19, and HCV datasets. As shown in Fig. 6, we evaluate each method’s performance in terms of accuracy, indexing/training time, and search/prediction time.

For COVID-19, XGBoost achieved the highest accuracy with very low prediction latency, while retrieval methods required much less index-construction time but slightly longer search times. For HCV, XGBoost again achieved the highest accuracy, although with substantially higher training cost, whereas HNSW provided comparable accuracy with much lower index-construction time. A similar pattern was observed for HPV, where XGBoost and Flat retrieval achieved nearly identical accuracy but differed considerably in training/indexing and prediction/search costs.

Overall, supervised DNABERT-based classifiers, particularly XGBoost, provided the best predictive performance and fast inference, but at the expense of higher training cost. Retrieval-based methods offered much cheaper index construction and greater operational flexibility, with IVF and HNSW providing the most favorable balance between accuracy and search efficiency. Compressed indexes reduced computational or storage requirements further, although the associated accuracy loss varied across datasets.

#### Memory usage analysis

**Fig. 7:**
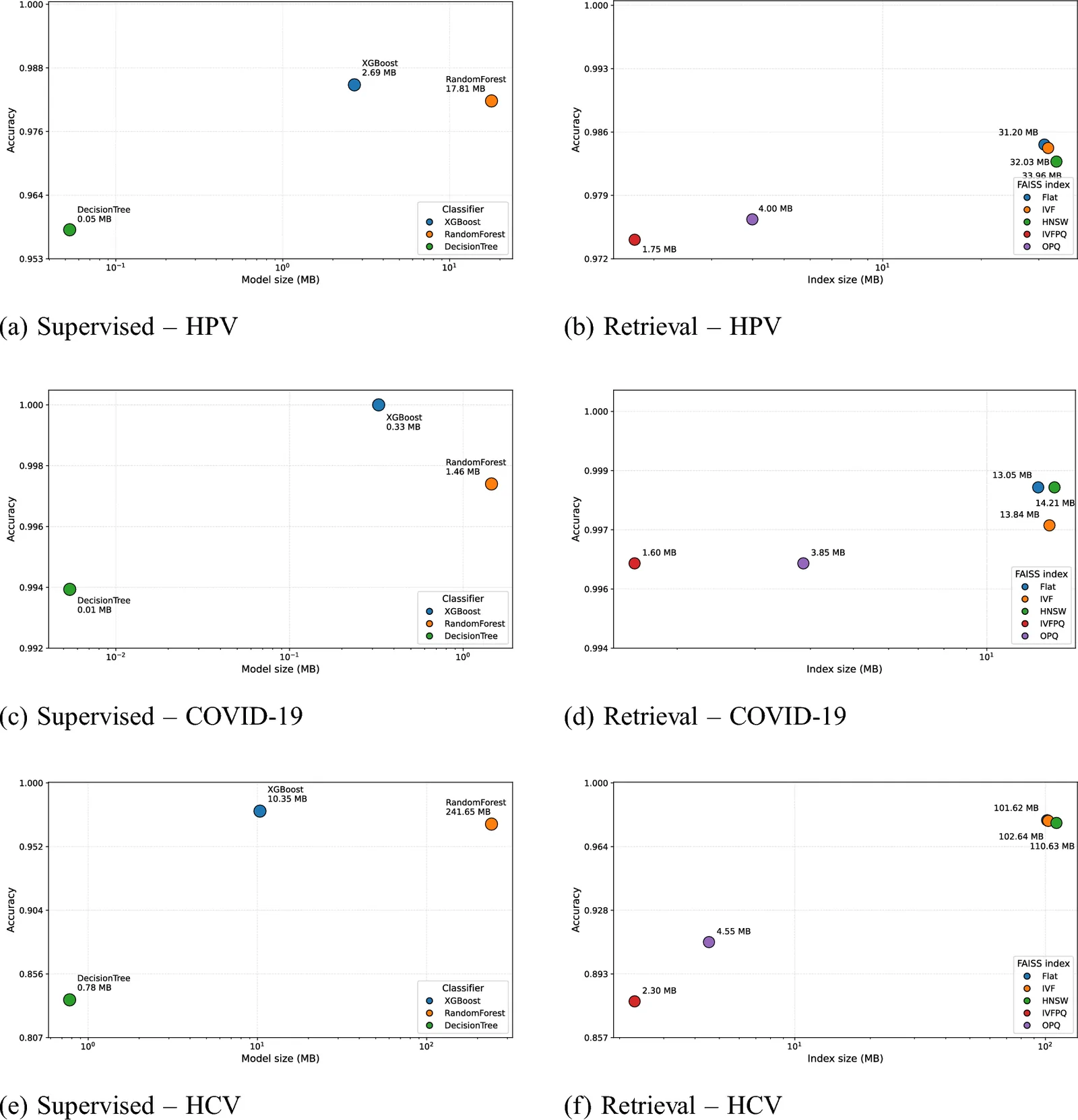
Comparison of classification accuracy and model/index size for supervised and retrieval-based classification using DNABERT embeddings across the HPV, COVID-19, and HCV datasets. Each row compares the supervised model size with the corresponding FAISS index size for the same dataset.

   In this experiment, we evaluate the trade-offs between model accuracy and memory usage across both supervised and similarity-based classification approaches using DNABERT embedding. As shown in Fig. 7, we compare how model or index size relates to predictive performance on the three genomic datasets. This analysis highlights the efficiency of different methods in terms of memory footprint relative to accuracy, and offers insights into which configurations provide the best balance for practical deployment.

Across all three datasets (COVID-19, HCV, HPV), supervised models demonstrate a favorable trade-off between accuracy and model/index size, with some models achieving high accuracy at modest memory footprints. For COVID-19, XGBoost achieves perfect accuracy (1.0) with a compact model size of only 0.33 MB, making it the most efficient model. In contrast, Random Forest yields slightly lower accuracy (0.997) at more than 4*× * the model size (1.46 MB). For HCV, the trade-off is more pronounced. XGBoost provides the highest accuracy (0.979) with a model size of 10.35 MB, while Random Forest comes close (0.969) but at a significantly larger size of 241.65 MB. For HPV, XGBoost again shows strong performance with high accuracy (0.985) and a model size of just 2.69 MB, outperforming Random Forest (0.982 at 17.81 MB).

In all datasets, Decision Tree offers the smallest model size (e.g., 0.01–0.78 MB) but suffers in accuracy, especially for HCV (only 0.836), indicating a clear trade-off between simplicity and performance.

For the similarity-based approach, a similar pattern is observed: simpler structures lead to smaller index sizes but lower accuracy. For COVID-19, all methods achieve high accuracy (*>0.996*), but compressed indexes like IVFPQ and OPQ reach this with much smaller memory footprints (1.6 MB and 3.85 MB) compared to Flat or HNSW (*≈ *13–14 MB). For HCV, the impact is more significant. Flat, IVF, and HNSW attain top accuracies (*≈ *0.979) with large indexes (*∼ *100 MB), whereas OPQ and IVFPQ reduce the size (2–5 MB) but at the cost of notable accuracy drops (e.g., OPQ at 0.912, IVFPQ at 0.878). For HPV, the pattern holds: Flat and IVF offer the highest accuracy (0.985, 0.984) with index sizes around 31–33 MB, while the smallest structure (IVFPQ at 1.75 MB) results in the lowest accuracy (0.974).

When comparing supervised and retrieval-based approaches, a consistent trend emerges: Supervised models, particularly XGBoost, offer a more favorable size-accuracy trade-off. For example, in COVID-19 and HPV, they achieve superior or equal accuracy with significantly smaller model sizes than the best retrieval indexes (over 13 MB). Retrieval-based methods, while slightly more memory-hungry, provide flexibility in terms of indexing and scalability. They remain competitive in accuracy, especially for COVID-19 and HPV, and their performance is tightly linked to the choice of index structure. For HCV, retrieval-based classification shows a sharper trade-off. Achieving comparable accuracy to supervised models requires much larger index sizes (*≈ *100 MB), whereas compressed retrieval methods suffer notable accuracy losses.

Overall, supervised learning offers better memory efficiency and performance, especially with XGBoost. Retrieval-based methods are viable alternatives when scalability or fast querying is prioritized, but their performance is more sensitive to the indexing strategy and dataset complexity.

#### Representation length effect

**Fig. 8:**
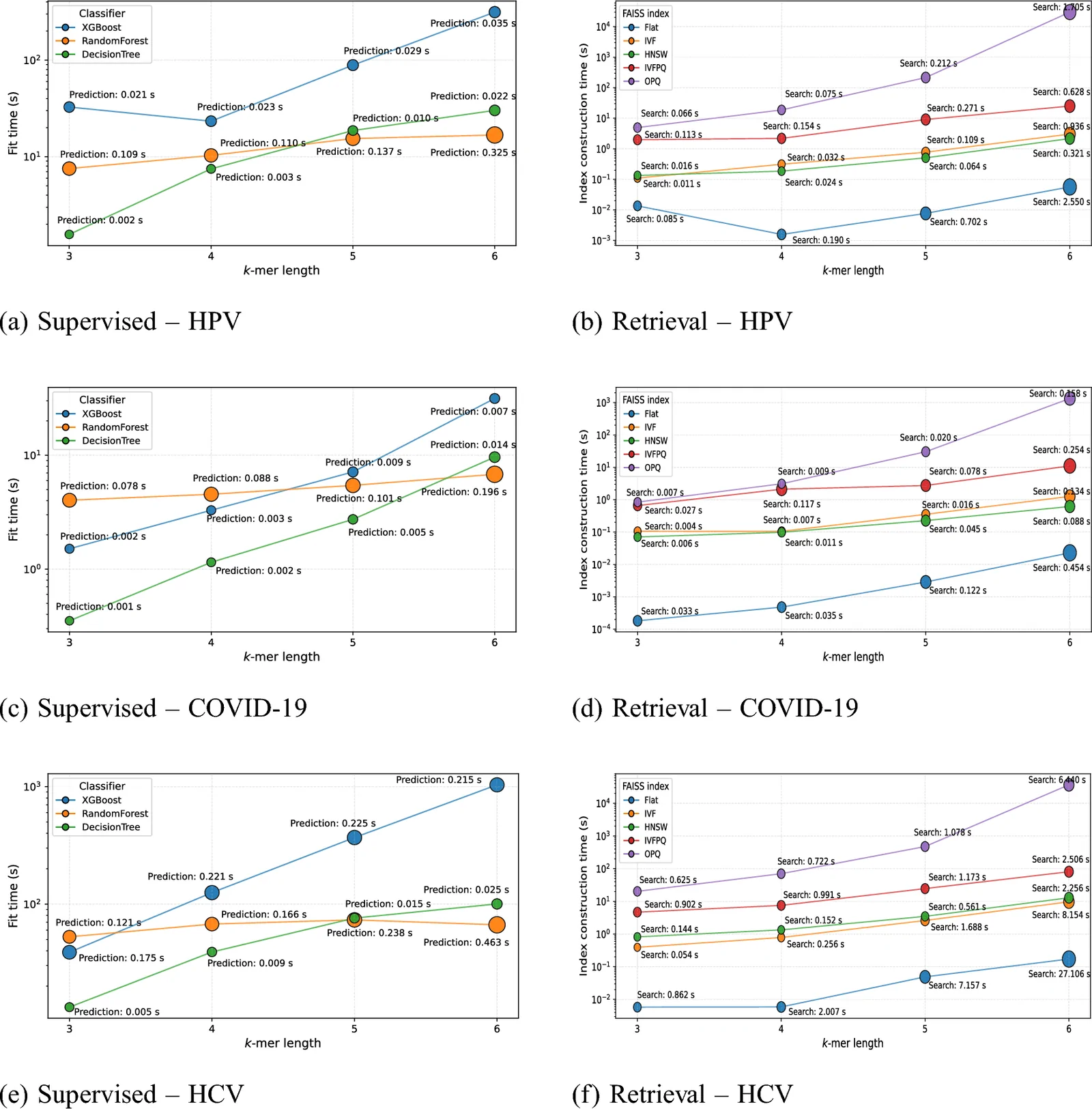
Comparison of computational time across different *k*-mer representation lengths for supervised and retrieval-based classification on the HPV, COVID-19, and HCV datasets. Each row compares the supervised and retrieval-based approaches for the same dataset.

In this experiment, we analyze how the length of the input representation impacts both the fit (indexing) time and the prediction (search) time for similarity-based and supervised classification tasks across the three datasets (Fig. 8). We compare supervised learning models (Random Forest, XGBoost, Decision Tree) with retrieval-based similarity search (Flat, IVF, HNSW, IVFPQ, OPQ) to highlight computational efficiency trends with respect to representation complexity.

For the HPV dataset, increasing the *k*-mer length leads to a noticeable rise in training time across both supervised and retrieval approaches. XGBoost’s training time shows the steepest growth among supervised models, reaching over 300 seconds for *k=6*. In contrast, Decision Tree remains lightweight even with higher k values.

On the retrieval side, indexing time increases sharply for OPQ and IVFPQ, particularly at *k=6*, while Flat indexing remains consistently low. Despite the computational cost, both approaches maintain high classification accuracy (*∼ *0.99), demonstrating the resilience of representation length for this dataset.

For the COVID-19 dataset, XGBoost has the steepest increase, rising from approximately 1.5 seconds at *k=3* to 31.5 seconds at *k=6*. Random Forest scales more moderately. The fit time increases from 4.0 seconds at *k=3* to 6.8 seconds at *k=6*. However, Decision Tree remains consistently the fastest to train.

The search time per evaluation remains low—typically in the millisecond range—and grows only mildly with *k*. Thus, in supervised learning, the primary computational cost of increasing *k*-mer length is incurred during training, not inference.

For similarity-based classification, Flat indexes are built in negligible time (e.g., $10^{-4}$–$10^{-2}$ s), but search latency grows with *k*, reaching 0.454 s at *k=6*. IVF and HNSW show a favorable balance, indexing within 1.26 s and search times around 0.1 s. However, IVFPQ and OPQ require more time to index (11 s and 1339 s respectively), though their search times remain competitive (0.254 s and 0.157 s).

A similar trend is observed for the HCV dataset, where the training time strongly depends on *k*-mer length. XGBoost grows rapidly, from 39 s at *k=3* to 1035 s at *k=6*. Random Forest shows a more moderate increase in training time, whereas Decision Tree is again the most efficient, starting at 13 s and reaching 100 s in training time.

For the retrieval approach, Flat indexing grows sharply, where indexing time grows from 0.005 s at *k=3* to 0.17 s at *k=6*, and the search time from 0.862 s at *k=3* to 27.106 s at *k=6*. IVF and HNSW maintain a better trade-off, with indexing times under 13 s and search times from 0.05 s to 8.15 s. IVFPQ reaches about 2.56 s in search time with index times up to 80 s, while OPQ becomes prohibitively expensive, with search time reaching approximately 6.440 s at *k=6* for only marginal gains in search efficiency.

Overall, increasing *k*-mer length sharply increases computational cost for both supervised and similarity-based methods, while bringing only modest accuracy gains. XGBoost and OPQ become particularly expensive, whereas Decision Tree, IVF, and HNSW offer a more favorable trade-off between efficiency and performance. This highlights a clear trade-off between representational richness and scalability.

**Fig. 9:**
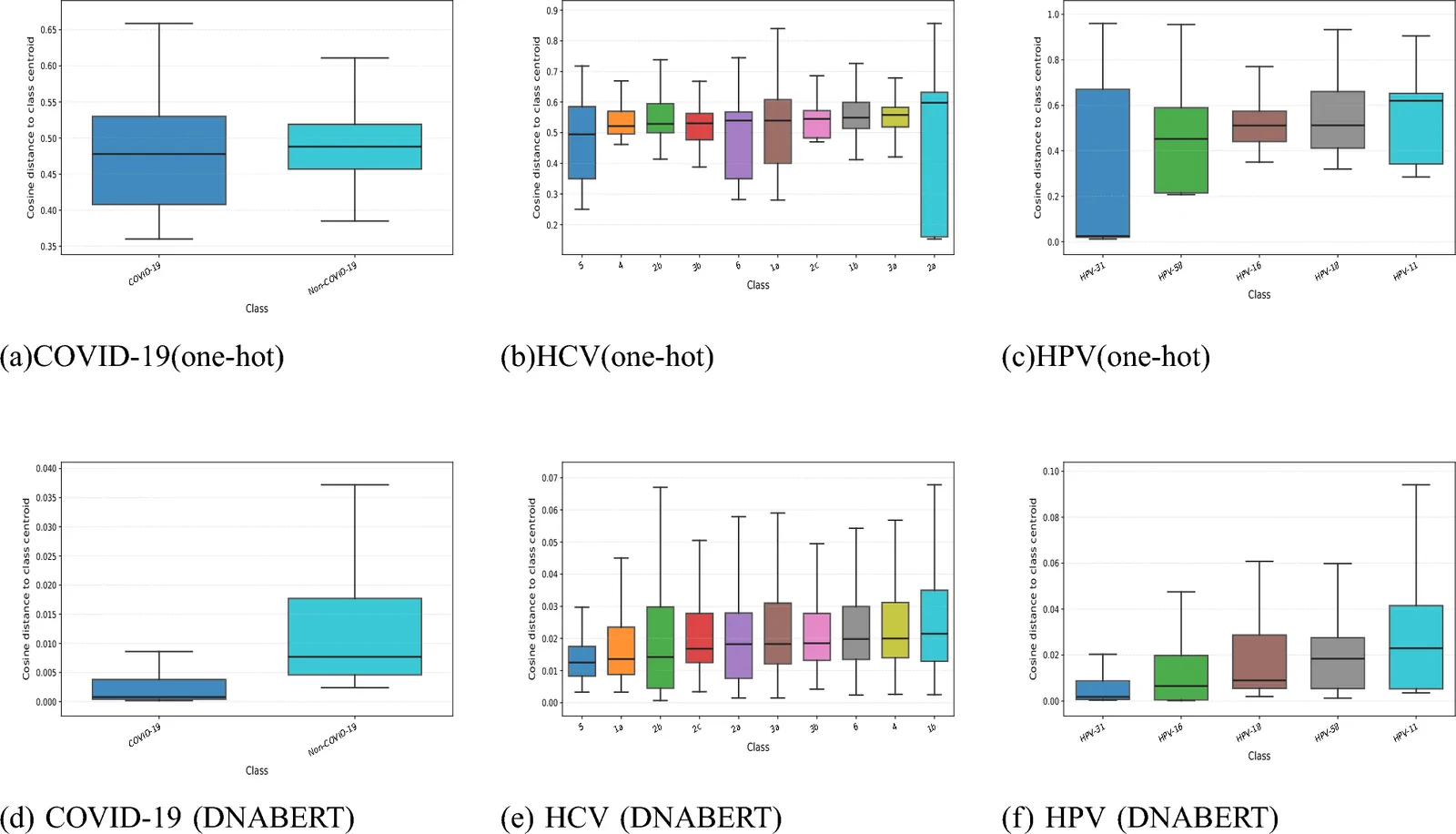
Per-class compactness: distance to own centroid for one-hot (top row) and DNABERT embeddings (bottom row).

#### Effect of sequence representation on intra-class distances

   In this experiment, we carry out an analysis comparing two encoders (DNABERT and one–hot) to study how the choice of representation affects the geometry of the embedding space. Since our similarity search relies on retrieving nearest neighbors under cosine similarity, it is important that sequences from the same class form compact clusters. To capture this effect, we examine the distribution of intra-class distances, defined as the cosine distance between each embedded sequence and the centroid (mean embedding) of its true class.

Figure 9 visualizes the intra-class distances for all classes and all datasets using boxplots. Across the COVID-19, HCV, and HPV datasets, DNABERT consistently produces much smaller intra-class distances: for most classes, the median distance to the class centroid is very close to zero and the spread within each box is narrow, indicating that samples of the same class are tightly grouped in the DNABERT space. In contrast, the one–hot representation yields substantially larger and more variable distances, with medians around the middle of the cosine distance range, showing that samples from the same class are far more dispersed.

Overall, the plots demonstrate that DNABERT embeddings induce substantially more compact class clusters than one–hot encodings in all three datasets. Because our similarity-search classifier aggregates labels from nearby points, having tight, well-formed clusters directly facilitates more reliable neighbor votes. The higher per-class compactness observed for DNABERT is therefore consistent with, and helps explain, its superior classification performance compared to the one–hot encoding.

## Conclusion

In this work, we presented a unified benchmark of supervised and retrieval-based methods for viral genomic sequence classification. The study systematically evaluated established sequence representations, supervised classifiers, and FAISS-based similarity-search indexes under a common experimental protocol. We compared one-hot, *k*-mer, FCGR, dna2vec, and DNABERT representations together with Random Forest, Decision Tree, XGBoost, and five FAISS index families across three case studies: HCV genotyping, COVID-19 discrimination, and HPV genotyping.

Among the supervised models, XGBoost combined with *k*-mer or FCGR representations, as well as DNABERT embeddings in the evaluated settings, provided strong classification performance, compact model sizes, and low prediction latency. Retrieval-based classification using Flat or IVF indexes often achieved comparable performance, particularly for the HPV and COVID-19 datasets, while allowing straightforward updates to the reference collection: encoded vectors from newly labeled sequences can be added without retraining a global supervised classifier. More strongly compressed indexes, including IVFPQ and OPQ, substantially reduced memory usage but incurred dataset- and representation-dependent accuracy losses, particularly for the HCV dataset.

Within the evaluated experimental setting, these findings show that supervised and retrieval-based methods should be regarded as complementary approaches. Supervised models may be preferable when the label space is stable and predictive accuracy, inference latency, or compact model size is the primary requirement. Retrieval-based methods may be useful when labeled reference collections are updated frequently or when predictions need to be accompanied by retrieved reference examples. This updateability is an operational property of the retrieval system and should not be interpreted as evidence of generalization to unseen genotypes, emerging variants, or temporally distinct sequences.

Although exact duplicate sequences were removed before partitioning, the random record-level splitting procedure did not explicitly prevent highly similar or near-duplicate sequences from appearing in both the training and test sets. Furthermore, the SARS-CoV-2 experiment represents a broad binary distinction between SARS-CoV-2 and other human coronaviruses and should not be interpreted as a lineage- or variant-level classification task.

Future work should extend the benchmark through similarity-aware partitioning, temporal splits, external validation datasets, more challenging lineage- and variant-level classification tasks, and metagenomic applications. Additional genomic language models should also be investigated. Evaluation across a broader range of datasets and application settings remains necessary as well.

## Supplementary Information

Supplementary Information 1.

Supplementary Information 2.

Supplementary Information 3.

## Data Availability

The accession numbers of the used sequences are available as supplementary material.

## Appendix A Implementation details and reproducibility

See Table 12.

**Table 12:** Main implementation and hyperparameter settings used for supervised and retrieval-based classification.

Data partitioning and evaluation	
Data partition	Stratified 80% training/20% testing; individual sequences used as the splitting unit; random seed 123; one fixed partition.

Dense sequence representations	
Embedding aggregation	Sequences were divided into sliding windows with 25% overlap, corresponding to $S=\lfloor 0.75W\rfloor$. Window embeddings were uniformly averaged to obtain one sequence-level representation, followed by row-wise $\ell _2$ normalization.
dna2vec	Pretrained encoder; window length *W=1024* nucleotides; stride *S=768*; batch size 16; 1,020-dimensional sequence representation.
DNABERT	Pretrained encoder with 6-mer tokenization; window length *W=515* nucleotides; stride *S=386*; batch size 16; 768-dimensional sequence representation.

Supervised classifiers	
Random Forest	300 trees; unrestricted maximum depth; all available CPU cores; random seed 123.
Decision Tree	Default scikit-learn hyperparameters; random seed 123.
XGBoost	400 estimators; learning rate 0.1; maximum depth 8.

Retrieval-based classification	
Vector preparation	Feature vectors stored as 32-bit floating-point arrays and $\ell _2$-normalized.
Neighbor selection	Number of retrieved neighbors evaluated over $k\in \{1,3,5\}$.
Voting rule	Similarity-weighted voting.

FAISS index configurations	
Flat	Exact exhaustive inner-product search.
IVF	256 inverted lists; 16 lists searched per query.
HNSW	*M=32* graph connections per node; efConstruction=200; efSearch=128.
IVFPQ	256 inverted lists; 16 probes; 16 product-quantization subspaces; 8 bits per subspace.
OPQ	64-subspace orthogonal transformation followed by IVFPQ with 16 product-quantization subspaces and 8-bit codes.

## Appendix B Additional classification metrics

### Appendix B.1 Macro-F1 scores for supervised classification

This section reports the Macro-F1 scores corresponding to the supervised classification experiments presented in Section Supervised classification performance. While accuracy is reported in the main text, Macro-F1 provides a class-balanced performance measure, which is particularly important for imbalanced datasets such as HCV and HPV (Tables 13, 14, 15, 16 and 17)

**Table 13:** Classification Macro-F1 scores using one-hot encoding across the three genomic datasets.

Model	HPV	COVID-19	HCV
XGBoost	0.934456	0.968186	0.955532
Random Forest	0.927379	0.972814	0.952458
Decision Tree	0.884846	0.947946	0.885623

**Table 14:** Classification Macro-F1 scores by model using k-mer encoding across the three genomic datasets.

Model	k-mer	HPV	COVID-19	HCV
XGBoost	3	0.970410	0.997267	0.973659
	4	0.974284	0.997268	0.984060
	5	0.973401	0.996358	0.986773
	6	0.976344	0.994540	0.986810
Random Forest	3	0.973216	0.999089	0.967366
	4	0.977533	0.997268	0.977686
	5	0.979738	0.993632	0.982858
	6	0.979491	0.995449	0.985940
Decision Tree	3	0.920451	0.990879	0.799405
	4	0.938556	0.992714	0.834721
	5	0.954274	0.989070	0.863164
	6	0.957414	0.989988	0.899714

**Table 15:** Classification Macro-F1 scores by model using FCGR encoding across the three genomic datasets.

Model	FCGR-*k*	HPV	COVID-19	HCV
XGBoost	3	0.9586	0.9973	0.9785
	4	0.9632	0.9964	0.9870
	5	0.9619	0.9964	0.9909
	6	0.9639	0.9954	0.9908
Random Forest	3	0.9665	0.9973	0.9696
	4	0.9659	0.9954	0.9808
	5	0.9692	0.9964	0.9864
	6	0.9715	0.9964	0.9894
Decision Tree	3	0.9108	0.9881	0.8183
	4	0.9291	0.9909	0.8326
	5	0.9333	0.9918	0.8657
	6	0.9469	0.9900	0.9000

**Table 16:** Classification Macro-F1 scores by model using dna2vec embeddings across the three genomic datasets.

Model	HPV	COVID-19	HCV
XGBoost	0.970286	0.992723	0.969970
Random Forest	0.958759	0.991812	0.918820
Decision Tree	0.890606	0.988151	0.638167

**Table 17:** Classification Macro-F1 scores by model using DNABERT embeddings across the three genomic datasets.

Model	HPV	COVID-19	HCV
XGBoost	0.961125	1.000000	0.972152
Random Forest	0.960998	0.997267	0.957251
Decision Tree	0.915681	0.993620	0.795161

### Appendix B.2 Macro-F1 scores for similarity-based classification

This section reports Macro-F1 scores for similarity-based classification using FAISS across different index types and encoding methods. These results complement the accuracy analysis presented in Section Similarity-based classification performance and provide additional insight into performance across classes (Tables 18, 19, 20, 21 and 22)

**Table 18:** Similarity-based Macro-F1 scores for one-hot representations across FAISS index types.

Index	HPV	COVID-19	HCV
Flat	0.915958	0.973628	0.947332
IVF	0.914188	0.973640	0.947725
HNSW	0.911292	0.972713	0.946301
IVFPQ	0.833317	0.948211	0.663639
OPQ	0.916779	0.964470	0.914488

**Table 19:** Similarity-based Macro-F1 scores for each FAISS index type and k-mer size. For each dataset, the Macro-F1 score and the *k*-NN value yielding the best score are reported.

Index	k-mer	HPV	COVID-19	HCV
Flat	3	0.976134 (*k=1*)	0.997268 (*k=1*)	0.982469 (*k=1*)
	4	0.978508 (*k=3*)	0.998178 (*k=1*)	0.988632 (*k=1*)
	5	0.982854 (*k=3*)	0.998178 (*k=1*)	0.989993 (*k=1*)
	6	0.983060 (*k=3*)	0.998178 (*k=1*)	0.990032 (*k=5*)
IVF	3	0.975775 (*k=5*)	0.997268 (*k=1*)	0.982438 (*k=1*)
	4	0.978782 (*k=3*)	0.998178 (*k=1*)	0.988335 (*k=1*)
	5	0.985124 (*k=3*)	0.998178 (*k=1*)	0.989916 (*k=1*)
	6	0.980966 (*k=5*)	0.998178 (*k=1*)	0.989870 (*k=5*)
HNSW	3	0.968855 (*k=1*)	0.997268 (*k=1*)	0.980573 (*k=1*)
	4	0.968599 (*k=1*)	0.998178 (*k=1*)	0.984320 (*k=1*)
	5	0.969460 (*k=1*)	0.998178 (*k=1*)	0.987814 (*k=1*)
	6	0.977179 (*k=1*)	0.998178 (*k=1*)	0.989092 (*k=1*)
IVFPQ	3	0.970662 (*k=3*)	0.997268 (*k=1*)	0.967066 (*k=5*)
	4	0.976300 (*k=1*)	0.998178 (*k=1*)	0.972204 (*k=5*)
	5	0.983064 (*k=1*)	0.998178 (*k=1*)	0.969336 (*k=5*)
	6	0.982210 (*k=1*)	0.998178 (*k=1*)	0.962099 (*k=1*)
OPQ	3	0.924405 (*k=3*)	0.997268 (*k=1*)	0.884723 (*k=5*)
	4	0.969127 (*k=5*)	0.998178 (*k=1*)	0.884428 (*k=3*)
	5	0.974592 (*k=3*)	0.998178 (*k=1*)	0.906366 (*k=1*)
	6	0.979260 (*k=3*)	0.998178 (*k=1*)	0.923570 (*k=3*)

**Table 20:** Similarity-based Macro-F1 scores for each FAISS index type and FCGR order *k*. For each dataset, the Macro-F1 score and the *k*-NN value yielding the best score are reported.

Index	FCGR-*k*	HPV	COVID-19	HCV
Flat	3	0.977201 (*k=1*)	0.997268 (*k=1*)	0.982088 (*k=1*)
	4	0.978344 (*k=3*)	0.998178 (*k=1*)	0.987764 (*k=1*)
	5	0.982576 (*k=3*)	0.998178 (*k=1*)	0.989210 (*k=1*)
	6	0.980814 (*k=1*)	0.998178 (*k=1*)	0.990081 (*k=1*)
IVF	3	0.975796 (*k=5*)	0.997268 (*k=1*)	0.982176 (*k=1*)
	4	0.976943 (*k=3*)	0.998178 (*k=1*)	0.987461 (*k=1*)
	5	0.983781 (*k=3*)	0.998178 (*k=1*)	0.989030 (*k=1*)
	6	0.980908 (*k=5*)	0.998178 (*k=1*)	0.990081 (*k=1*)
HNSW	3	0.969825 (*k=1*)	0.997268 (*k=1*)	0.980957 (*k=1*)
	4	0.965249 (*k=1*)	0.998178 (*k=1*)	0.983436 (*k=1*)
	5	0.972098 (*k=1*)	0.998178 (*k=1*)	0.988216 (*k=1*)
	6	0.974580 (*k=3*)	0.998178 (*k=1*)	0.989370 (*k=1*)
IVFPQ	3	0.972736 (*k=5*)	0.997268 (*k=3*)	0.965208 (*k=5*)
	4	0.973968 (*k=1*)	0.998178 (*k=1*)	0.958470 (*k=5*)
	5	0.980360 (*k=1*)	0.998178 (*k=1*)	0.957185 (*k=5*)
	6	0.981712 (*k=3*)	0.998178 (*k=1*)	0.960736 (*k=5*)
OPQ	3	0.969213 (*k=5*)	0.998178 (*k=1*)	0.959005 (*k=3*)
	4	0.977098 (*k=3*)	0.998178 (*k=1*)	0.962737 (*k=3*)
	5	0.978999 (*k=5*)	0.998178 (*k=1*)	0.970065 (*k=3*)
	6	0.979163 (*k=3*)	0.998178 (*k=1*)	0.973073 (*k=5*)

**Table 21:** Similarity-based Macro-F1 scores for each FAISS index type using dna2vec embeddings. The corresponding optimal *k*-NN neighborhood size is shown in parentheses.

Index	HPV	COVID-19	HCV
Flat	0.976984 (*k=1*)	0.997264 (*k=1*)	0.982151 (*k=1*)
IVF	0.976984 (*k=1*)	0.997264 (*k=1*)	0.981734 (*k=1*)
HNSW	0.976984 (*k=1*)	0.995441 (*k=1*)	0.995441 (*k=1*)
IVFPQ	0.967806 (*k=3*)	0.991805 (*k=1*)	0.902288 (*k=5*)
OPQ	0.965968 (*k=5*)	0.991801 (*k=1*)	0.959756 (*k=5*)

**Table 22:** Similarity-based Macro-F1 scores for each FAISS index type using DNABERT embeddings. The corresponding optimal *k*-NN neighborhood size is shown in parentheses.

Index	HPV	COVID-19	HCV
Flat	0.973465 (*k=3*)	0.998178 (*k=1*)	0.970760 (*k=1*)
IVF	0.972665 (*k=3*)	0.997267 (*k=1*)	0.970671 (*k=1*)
HNSW	0.959667 (*k=1*)	0.997266 (*k=1*)	0.970016 (*k=1*)
IVFPQ	0.957970 (*k=3*)	0.997266 (*k=1*)	0.848081 (*k=5*)
OPQ	0.961704 (*k=1*)	0.995443 (*k=3*)	0.886720 (*k=5*)
